# Light absorption enhancement in ultrathin film solar cell with embedded dielectric nanowires

**DOI:** 10.1038/s41598-020-74453-7

**Published:** 2020-10-16

**Authors:** Mahmoud A. Elrabiaey, Mohamed Hussein, Mohamed Farhat O. Hameed, Salah S. A. Obayya

**Affiliations:** 1 Centre for Photonics and Smart Materials, Zewail City of Science and Technology, October Gardens, 6th of October City, Giza, 12578 Egypt; 2 Department of Physics, Faculty of Science, Ain Shams University, Abbassia, 11566 Cairo Egypt; 3 Nanotechnology and Nanoelectronics Engineering Program, University of Science and Technology, Zewail City of Science and Technology, October Gardens, 6th of October City, Giza, 12578 Egypt; 4 Mathematics and Engineering Physics Department, Faculty of Engineering, Mansoura University, Mansoura, 35516 Egypt

**Keywords:** Nanoscience and technology, Optics and photonics

## Abstract

A novel design of thin-film crystalline silicon solar cell (TF C-Si-SC) is proposed and numerically analyzed. The reported SC has 1.0 µm thickness of C-Si with embedded dielectric silicon dioxide nanowires (NWs). The introduced NWs increase the light scattering in the active layer which improves the optical path length and hence the light absorption. The SC geometry has been optimized using particle swarm optimization (PSO) technique to improve the optical and electrical characteristics. The suggested TF C-Si-SC with two embedded NWs offers photocurrent density (${J}_{ph}$) of 32.8 mA cm^−2^ which is higher than 18 mA cm^−2^ of the conventional thin film SC with an enhancement of 82.2%. Further, a power conversion efficiency of 15.9% is achieved using the reported SC.

## Introduction

The solar power, is considered as the best abundant and non-polluting renewable energy source. Due to its abundance, non-toxicity and mature fabrication technology, silicon solar cell (SC) shares 90% from photovoltaic market investment^1^. The power conversion efficiency (PCE) of crystalline silicon (C-Si) SCs has reached the plateau region and its highest value approaches the theoretical limit of 27.6%^2^. To reduce the cost per watt, new generations of SCs have been introduced^1,2^. Thin film (TF) Si SC with active layer thickness of 1 µm is an economical solution for solar energy harvesting with low cost^1^. However, TF technology suffers from lower absorption coefficients at longer wavelengths. Further, the thickness is small to trap the light through the active layer. Therefore, the absorption and power conversion efficiencies will be reduced. In order to increase the light path length inside the active layer, several nanostructures have been employed such as surface texture^1,3,4^, plasmonic nanoparticles^1^, and nanowire^5^.

Recently, the dielectric nanoparticles are used to improve the optical absorption and the generated photocurrent in TF C-Si-SCs^6–10^. The scattering cross sections of the dielectric nanoparticles are smaller than those of plasmonic nanoparticles. However, the dielectric nanoparticles have an incomparable low light absorption than that of plasmonic materials. Moreover, the active layer with plasmonic nanoparticles suffers from electrical degradation, owing to the plasmonic high surface recombination velocity (SRV) (10^6^ cm s^−1^) at the Si/plasmonic interface^7^. Nunomur et al*.*^6^ have introduced tandem SC with embedded SiO_2_ nano-spheres with an enhancement of 16% in the short-circuit current density (${J}_{sc}$). Mopourisetty et al*.*^7^ have reported an electrical study for 1 µm TF C-Si-SC with surface recombination losses at a Si/SiO_2_ interface with a ${J}_{sc}$ of 11.9 mA cm^−2^. Yang et al.^8^ have introduced 2 µm TF C-Si-SC with partially embedded dielectric sphere with a photocurrent density (${J}_{ph}$) and ${J}_{sc}$ of 30.09 mA cm^−2^ and 28.6 mA cm^−2^, respectively. Further, Nagel et al*.*^9^ have improved the light trapping using embedded silicon dioxide (SiO_2_) nanospheres in TF-SC. Such a structure offers an absorption enhancement of 23.4% relative to the conventional SC.

In this work, we propose and analyze a new design approach for enhancing the efficiency of TF-SCs with a thickness of 1 µm. The suggested technique is based on lateral scattering using dielectric nanowires embedded within the active layer. Such dielectric nanowires are nearly lossless with high cross-sectional scattering along their length which can improve the SC efficiency. In this study, the SiO_2_ is used as a scatter element due to the large index contrast with the Si material (n = 3.6). Additionally, the SiO_2_ is almost perfectly lossless within the visible spectrum. Under the right circumstances, Si/SiO_2_ interfaces may have extremely low interface recombination velocities and dangling bond densities. Further, the growth of SiO_2_ NWs can be implemented using oxidation process of Si^10^. The modified TF-SC with two embedded dielectric NWs offers power conversion efficiency (PCE) of 15.9% and an optical absorption enhancement of 82.2% over the conventional planer TF SC.

## Simulation strategy

**Figure 1:**
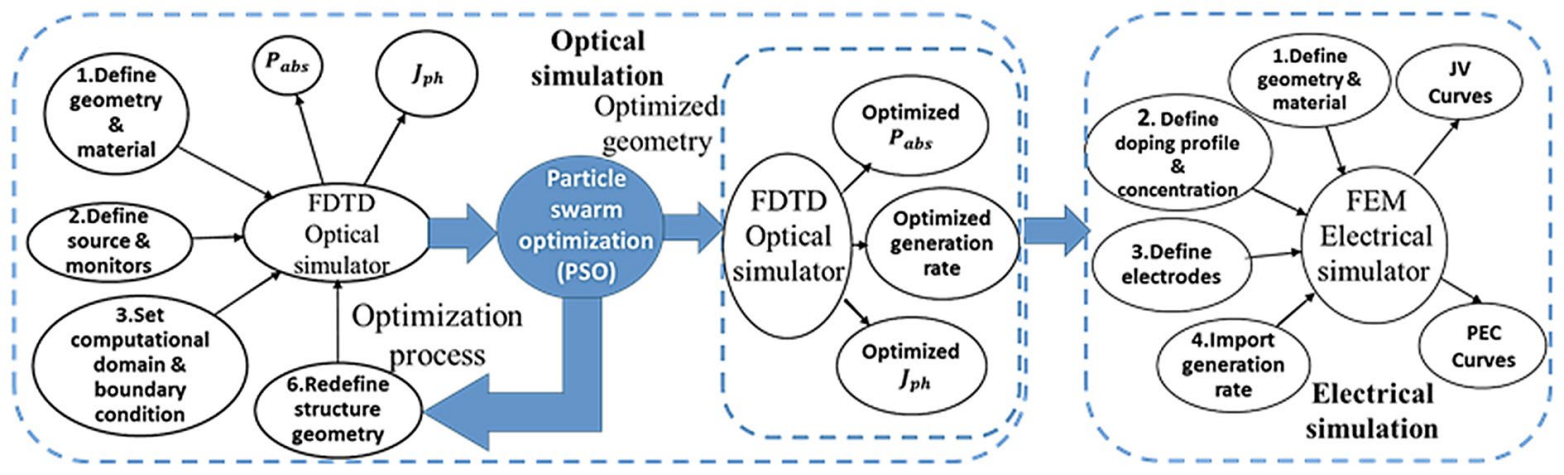
Simulation strategy of the proposed design using optical FDTD and electrical FEM simulators.

Figure 1 shows the simulation strategy for the SC characterization. First, optical studies are carried out by using 3D finite-difference time-domain (FDTD) via Lumerical software package to calculate the optical absorption (${P}_{abs}$) and ${J}_{ph}$^11^. To estimate the absorption capability of the TF C-Si-SCs, ${J}_{ph}$ is calculated under air mass 1.5 (AM 1.5 G) solar spectrum. To further enhance the ${P}_{abs}$ and ${J}_{ph}$ of the reported design, the geometrical parameters are optimized using particle swarm optimization (PSO) technique^12^. In this investigation the ${J}_{ph}$ is used as a fitness function of the PSO algorithm to quantify the broadband absorption capability of the suggested TF C-Si-SC^5^:

1 $${J}_{ph}=\frac{e}{hc}\underset{300}{\overset{1100}{\int }}\lambda {\phi }_{AM1.5}(\lambda ){P}_{abs}\left(\lambda \right)d\lambda$$

where $e$ is the electron unit charge, h is the Plank constant, $c$ is the light speed in vacuum, ${\Phi }_{AM1.5}$ is the solar spectral irradiance AM 1.5 as obtained from the NREL database^2^ and ${P}_{abs}$ is the optical absorption spectrum of the C-Si material.

Next, the generation rate of the optimal design is imported into the electrical simulator to quantify the photo-generated electron–hole pairs that can be collected and contributed to the output electrical power. The electrical model is used to calculate both the PCE and ${J}_{sc}$ using finite element method (FEM) via Lumerical device simulation package^11^.The FEM package solves coupled nonlinear equations of semiconductor (drift–diffusion equations and Poisson continuity) to obtain the power conversion efficiency (PCE) of the reported design^13–15^. The PCE can be calculated according to the following equation^7^:

2 $$PEC=\frac{F.F \times {J}_{sc}\times {V}_{oc}}{{P}_{in}}$$

where ${P}_{in}$ is the incident power at AM 1.5, $F.F$ is the fill factor defined as ($F.F= {P}_{max}/{J}_{sc}\times {V}_{oc}$), ${V}_{oc}$ is the open-circuit voltage, and ${P}_{max}$ is the maximum power.

## Design consideration and numerical results

**Figure 2:**
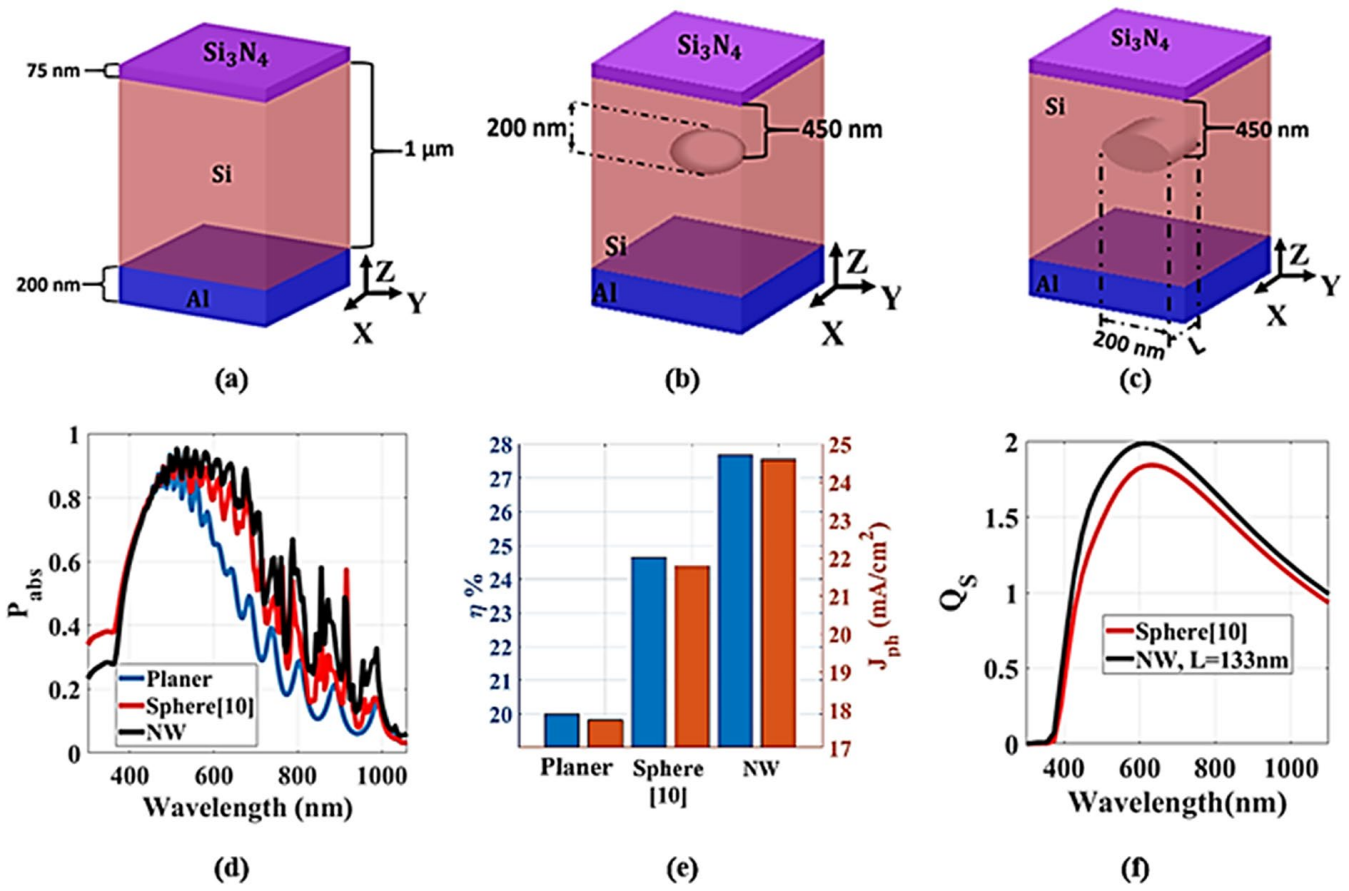
Schematic diagram of (**a**) planer SC, (**b**) SC with embedded dielectric sphere, and (**c**) SC with embedded dielectric NW of length (L). (**d**) The power absorption of the planar TFSC and TFSC with dielectric sphere and TF-SC with dielectric SiO_2_ NW, (**e**) the ${J}_{ph}$ and the absorption efficiency ($\upeta$) for the studied designs, and (**f**) the scattering efficiency of the SC with embedded SiO_2_ sphere^9^ and NW of the same volume at the same depth. This image is created by Lumerical 2020a, FDTD Solver Version 8.23.2305, https://www.lumerical.com (license number—12802) released to Zewail City of Science and Technology, Giza, Egypt.

Figure 2a–c show the schematic diagram of the planar TF-SC, TF-SC with embedded dielectric sphere and embedded nanowire, respectively. The embedded dielectric (SiO_2_) sphere and NW have the same volume and are positioned at 450 nm from the surface. The sphere and the NW have the same diameter of 200 nm as shown in Fig. 2b,c. Further, the nanowire has a length (L) of 133 nm. The proposed design is optically simulated using computational domain of 750 nm × 750 nm with height of 3 µm with minimum mesh size of 8.0 nm. In addition, periodic boundary conditions are used in the x and y-directions to mimic the effects of an infinitely periodic unit cell. However, the boundary condition along z axis is a perfect matched layer (PML). The suggested design is excited from the top by a plane wave with wavelength range from 300 to 1100 nm. The material refractive indices of silicon and silicon dioxide are predefined in the Lumerical material database based on Palik’s model^16^.

The TF-SC design with embedded dielectric (SiO_2_) sphere is previously published in^9^. The power absorption of the planar TF-SC, TF-SC with embedded dielectric sphere^9^ and embedded NW is shown in Fig. 2d. It can be seen from this figure that the TF-SC with dielectric scatter has higher absorption than the baseline TF-SC counterpart. Figure 2e illustrates the ${J}_{ph}$ and the absorption efficiency ($\eta$) for the three studied designs. It can be seen from this figure that the TF-SC with sphere and NW scatters have ${J}_{ph}$ and $\eta$ higher than that of the conventional base line counterpart. The ${J}_{ph}$ for the planar TF-SC and TF-SC with embedded dielectric sphere and nanowire are equal to 17.73, 21.9, and 24.6 mA cm^−2^, respectively. Additionally, $\eta$ of the planar TF-SC and TF-SC with embedded dielectric sphere and nanowire are equal to 20%, 24.65%, and 27.674%, respectively. This enhancement is attributed to the cylindrical geometry of the NW which has a higher scattering efficiency than that of the spherical geometry by 10.9% especially at λ ≥ 500 nm as shown in Fig. 2f. The scattering efficiency (${Q}_{s}$) is defined as the ratio between scattering cross-section of a dielectric particle and the physical cross-sectional area^9^. Therefore, higher scattering is achieved for the incident light inside the active layer by the cylindrical NWs. Consequently, the absorption and ${J}_{ph}$ are enhanced. It is also expected that the two embedded NWs can further improve the light absorption.

**Figure 3:**
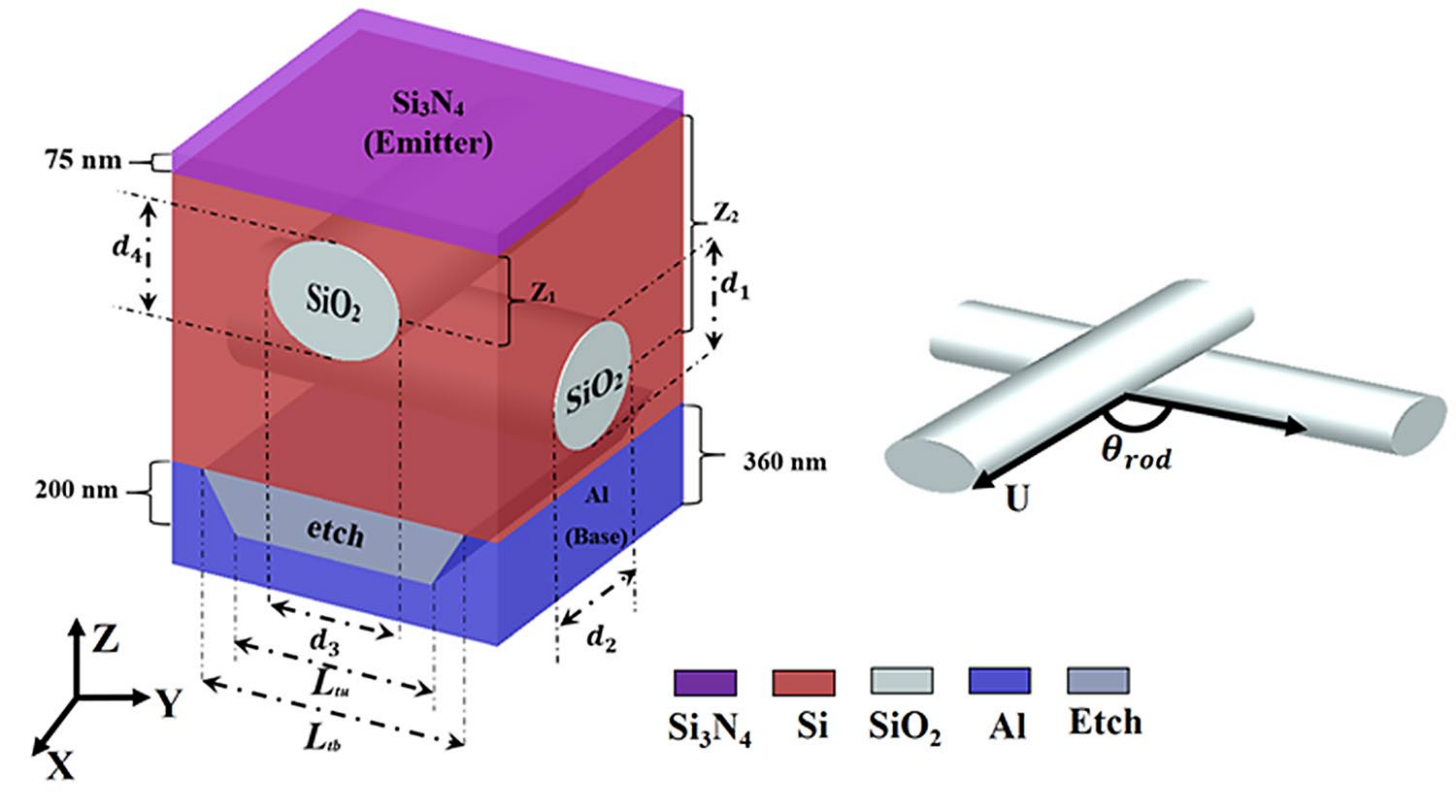
Schematic diagram of the proposed design with two dielectric nanowires (NWs). This image is created by Lumerical 2020a, FDTD Solver Version 8.23.2305, https://www.lumerical.com (license number—12802) released to Zewail City of Science and Technology, Giza, Egypt.

Next, the coupled optical/electrical modeling techniques are used to explore the cell performance of the proposed SC with three dielectric embedded elements (sphere, single NW, and two NWs) and compered with the conventional baseline SC. These designs are optically simulated using computational domain of $1\; \mathrm{\mu m}\times 1 \;\mathrm{\mu m}$ with height of 3 µm with minimum mesh size of 8.0 nm. Figure 3 shows 3D schematic diagram of the reported 1.0 µm TF C-Si-SC with two embedded nanowires. The suggested design has an antireflection coating of Si_3_N_4_ with a thickness of 75 nm^9^. Further, a trapezoidal grating is used as a back reflector with upper and lower bases of *L*_*tu*_ and *L*_*tb*_*,* respectively. The two embedded NWs have an elliptical shape with minor and major diameters of *d*_*1*_, *d*_*2*_, *d*_*3*_, and *d*_*4*_, respectively. The NWs are added in the active layer at a depth of Z_1_ and Z_2_ from the surface of the active layer with rotation angle of Θ_rod_ as shown in Fig. 3. In order to obtain the optimal design dimensions with maximum photocurrent density, the PSO technique is employed for the two embedded elements (single NW and two NWs). Table 1 summarizes the initial and optimized parameters of the reported design with one NW and two NWs cases. There are six optimization parameters which are minor, major diameters, the distance from the surface of the active layer and center of nanowire (${Z}_{NW}$), rotation angle of Θ_rod_ and the upper (*L*_*tu*_) and lower (*L*_*tb*_) bases of the trapezoid back grating etching. For single NW, Θ_rod_ is the angle between the NW and the x-axis while for the two NWs Θ_rod_ represents the angle between them. The initial and optimized geometrical parameters are listed in Table 1.

**Table 1:** The initial and optimized geometrical parameters of the proposed SC with embedded SiO_2_ NWs.

Parameters	Decision space		Optimum values	
	From	To	1 NW	2 NWs
${\mathrm{d}}_{1}$	200 nm	400 nm	200 nm	200 nm
${\mathrm{d}}_{2}$	200 nm	400 nm	302 nm	200 nm
${\mathrm{d}}_{3}$	200 nm	400 nm	-	248 nm
${\mathrm{d}}_{4}$	200 nm	400 nm	-	200 nm
Θ_rod_	0°	90°	20.8°	72.62°
Z_1_	−200 nm	200 nm	100 nm	336 nm
Z_2_	−200 nm	200 nm	-	536 nm
*L*_*tu*_	0	900 nm	900 nm	900 nm
*L*_*tb*_	0	900 nm	872 nm	900 nm

### Optical characterization

**Figure 4:**
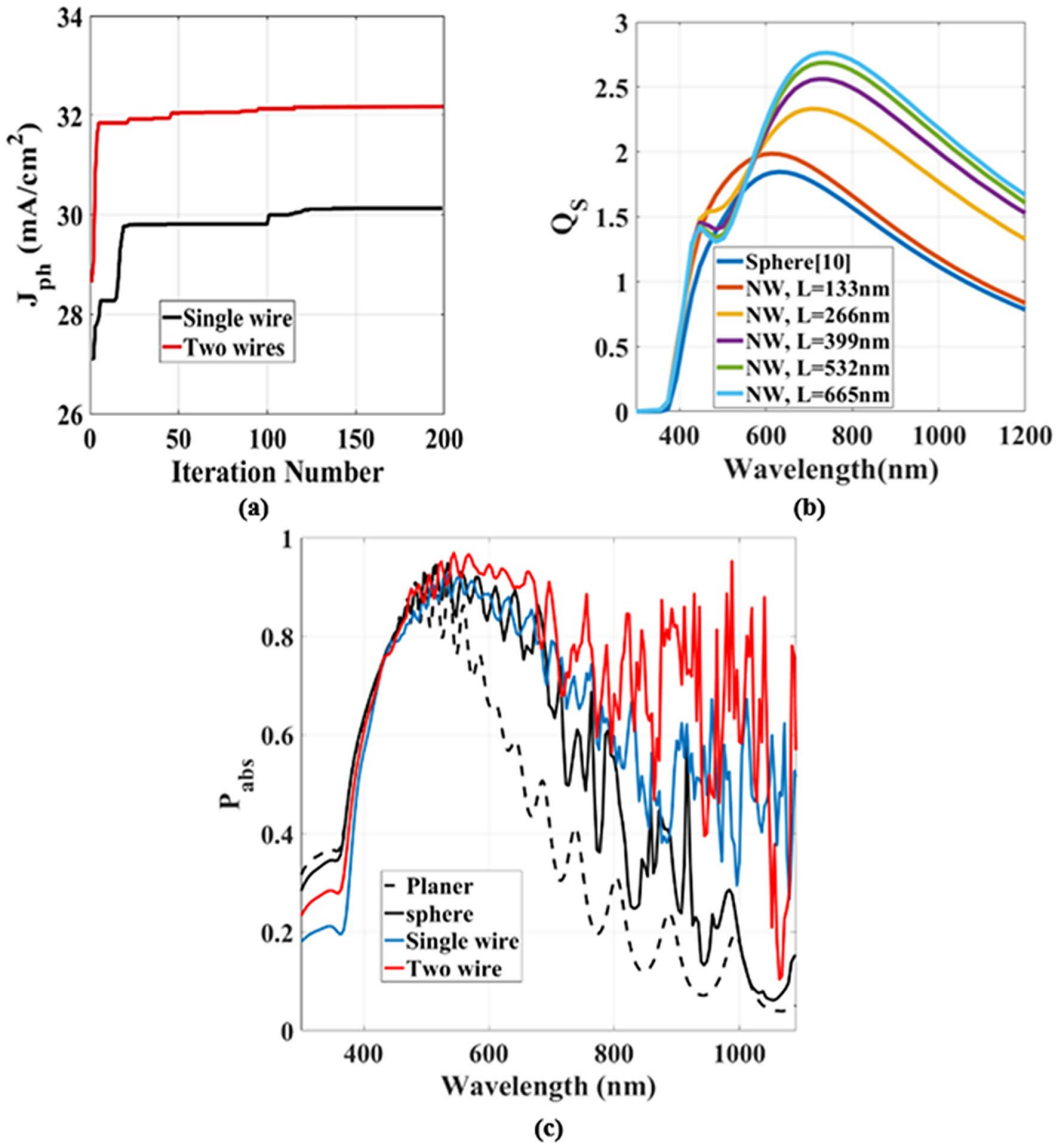
(**a**) The FOM trend of the PSO optimization for the single NW and the two NWs design, (**b**) the scattering efficiency as a function of the NW length (L), and (**c**) the absorption spectra of planer TF-SC (dotted line), TF-SC with embedded sphere^9^ (black line), and proposed design with single NW (blue line), and with two NWs (red line).

Figure 4a shows the optimization performance for the ${J}_{ph}$ of SCs with single/double NWs versus the iterations number. The optimized design offers ${J}_{ph}$ of 30.2 mA cm^−2^ and 32.8 mA cm^−2^, respectively which exceed the Lambertian limit for 1.0 µm TF C-Si-SC^17^. This enhancement is mainly attributed to the presence of embedded NWs as dielectric scatters. Therefore, an enhancement ratio of 67% and 82.2% respectively are achieved over the conventional planer TF C-Si-SC. It may be also seen from this figure that the optimizer has fast convergence with smaller iteration numbers using two NWs. The improvement in ${J}_{ph}$ is due to cylindrical geometry of the NWs that offers a degree of freedom through NW length (L) variation. Figure 4b shows the scattering efficiencies of the embedded dielectric sphere and NWs with multiple lengths ($L, \;2\times L, \;3\times L, \;4\times L, \;\mathrm{and} \;5\times L$). The dielectric elements have a diameter of 200 nm at a depth of 450 nm beneath the C-Si/Si_3_N_4_ interface^9^. The scattering efficiency is improved by increasing the length L especially at λ ≥ 500 nm. This enhancement is attributed to the NW length (characteristic length) which increases the scattering in the horizontal direction compared to spherical counterpart.

Figure 4c shows the ${P}_{abs}$ of the conventional planer TF C-Si-SC (baseline), TF C-Si-SC with embedded SiO_2_ nano-spheres^9^, single SiO_2_ NWs, and two SiO_2_ NWs. It may be seen from this figure that the SiO_2_ scatter will enhance the optical absorption compared to the planar reference SC device. As λ increases further than 450 nm, ${P}_{abs}$ of the planar device is decreased due to the low absorption coefficient of C-Si material at mid and high wavelength band^8,9^. However, by introducing the embedded NWs, the absorption enhancement is increased rapidly in the mid and high wavelength band owing to the geometry and configuration of the nanowires. Additionally, due to the improved impedance mismatch between the air and C-Si layer, more scattering occurs through the active layer with more light absorption. The ${J}_{ph}$ of the planer conventional TF-SC, TF-SC with embedded nano-spheres, one and two NWs are 18.3, 25.9, 30.2 and 32.8 mA cm^−2^, respectively.

**Figure 5:**
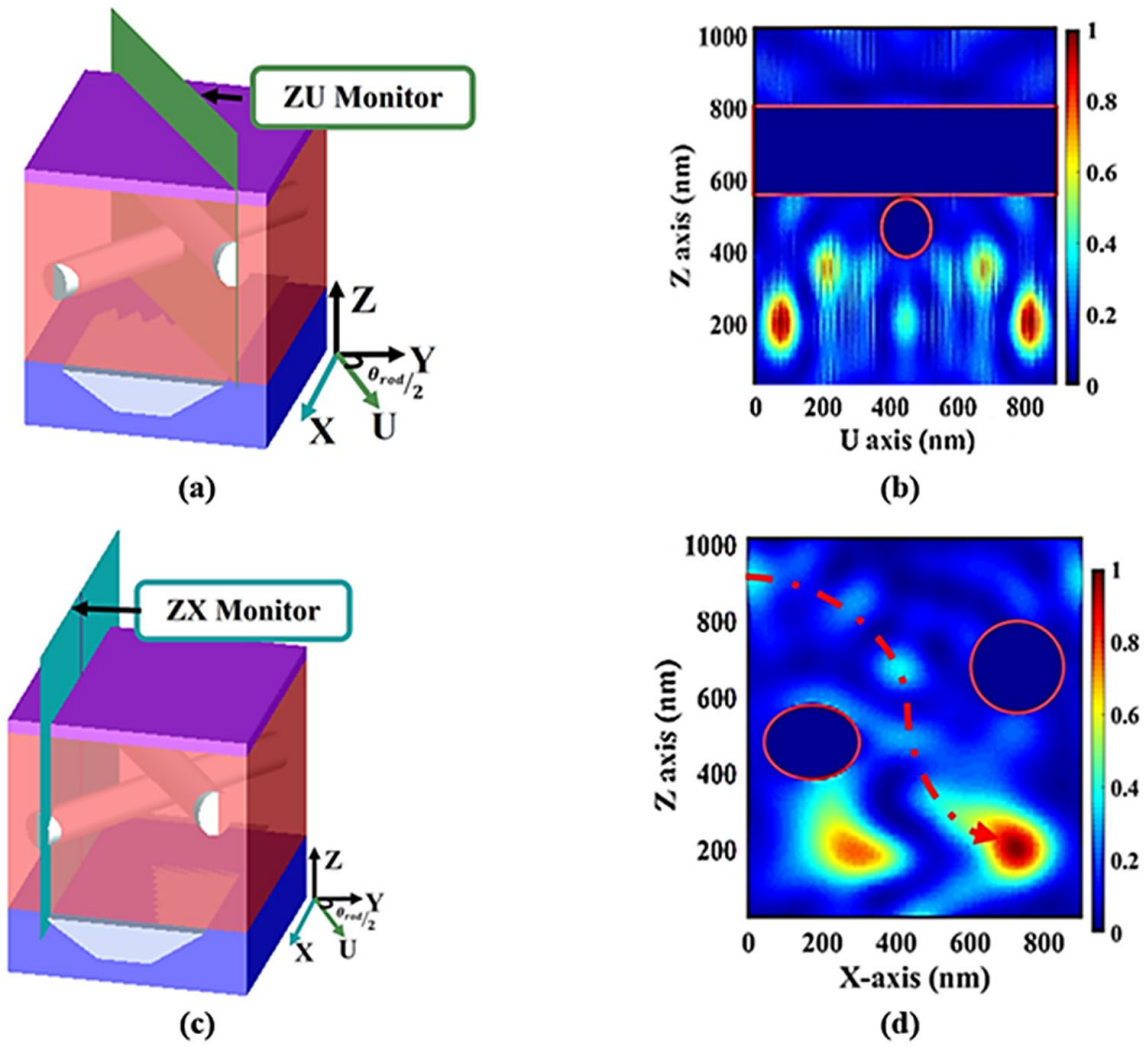
(**a**) Schematic diagram of proposed design with two NWs at the diagonal plane (ZU), (**b**) the normalized absorption distributions at cross section plane (ZU), (**c**) schematic diagram of the suggested design with two dielectric nanowires (NWs) at the side plane (ZX), and (**d**) the normalized absorption distributions for the two NWs at cross sectional plane (ZX). This image is created by Lumerical 2020a, FDTD Solver Version 8.23.2305, https://www.lumerical.com (license number—12802) released to Zewail City of Science and Technology, Giza, Egypt.

Figure 5b illustrates the normalized absorption distribution at cross sectional plane ZU shown in Fig. 5a. It may be seen that the light is highly trapped and absorbed below the NWs. For further investigation, another monitor is placed along the side plane (ZX) for the NWs as shown in Fig. 5c. The normalized absorption distributions at the edges of the TF C-Si-SCs is described in Fig. 5d where the light path is plotted red dotted line. Such figures confirm that the presence of NWs produces volume light scattering in the active layer which improves the optical absorption by tuning the scattering mean free path to the material absorption length.

**Figure 6:**
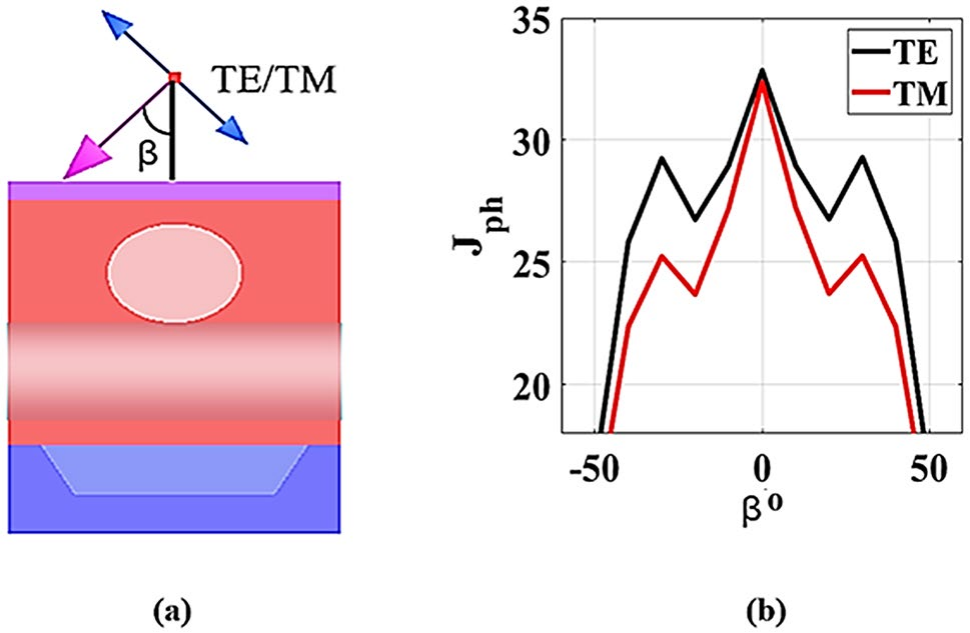
(**a**) Schematic diagram of the optimized design with NWs with an oblique incidence; and (**b**) the angular photocurrent response under TE and TM incidences for the two NWs C-Si-SCs. This image is created by Lumerical 2020a, FDTD Solver Version 8.23.2305, https://www.lumerical.com (license number—12802) released to Zewail City of Science and Technology, Giza, Egypt.

Figure 6a shows a schematic diagram of the reported SC with embedded two NWs under the excitation of transverse electric (TE) and transverse magnetic (TM) modes. In this study, the incident angle (β) for both excitation modes is swept from − 50° to 50°. Figure 6b shows the photon current density ${J}_{ph}$ versus the incidence angle for TE and TM polarizations. It may be seen from Fig. 6b that the ${J}_{ph}$ for TE and TM modes are nearly the same at normal incidence (β = 0°) and is equal to 32.9 mA cm^−2^. Additionally, a second maximum of ${J}_{ph}$ is achieved at β = 30° which is equal to 29.5 and 25.4 mA cm^−2^ for TE and TM excitations, respectively. The angular photocurrent response can be explained by the polarization angle effect. At the incident angle (β) = 0°, the light is nearly fully transmitted into the absorber without any reflection^18^. At β = 30°, the absorption enhancement is due to the increment of the optical path length in addition to the angular scattering from the two NWs. The difference between the ${J}_{ph}$ according to the TE and TM modes is due to the asymmetry of the two NWs in y-axis and x-axis. In this context, the tilted incident angle will increases the influence of the asymmetry of the NWs^18^.

### Electrical characterization

**Figure 7:**
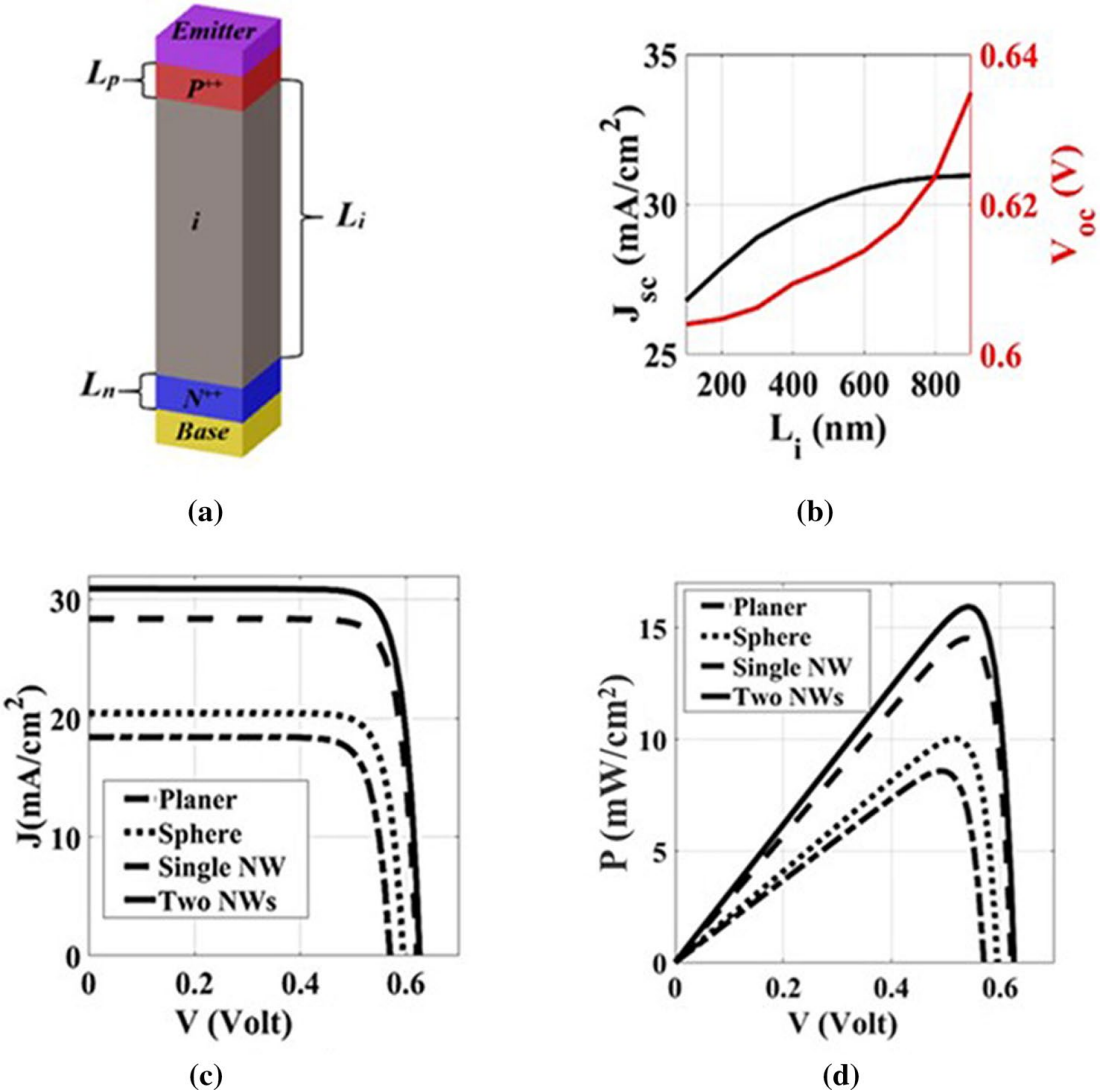
(**a**) The p—i—n axial doping, (**b**) the ${\mathrm{J}}_{\mathrm{sc}}$ and ${\mathrm{V}}_{\mathrm{oc}}$ dependence on the L_i_, (**c**) J–V characteristics of the C-Si TF planer and with different embedded dielectric elements: sphere, single NW, and two NWs, and (**d**) power density curves for C-Si TF planer and with different embedded dielectric elements: sphere^9^, single NW, and two NWs.

Figure 7a shows the schematic diagrams of the reported TF-SC with p-i-n doping (p-type/intrinsic/n-type) mounted on Si substrate. The proposed design utilizes the silicon nitrate and aluminum as the emitter and base electrodes, respectively as shown in Fig. 7a. In this study, the doping concentrations of P^+^ and N^+^ regions are equal to 5 × 10^17^ and 1 × 10^19^ cm^−3^, respectively^8,19^. However, the doping P^+^ and N^+^ regions are on the front and rear part of the SCs with thicknesses of L_p_ and L_n_, respectively. Also, the carrier lifetimes of P^+^, N, and N^+^ region are 10, 1000, and 5 μs, respectively. The surface recombination velocity is 2 × 10^5^ cm s^−1^^8^. Additionally, the Auger coefficients for electrons and holes are 9.9 × 10^–32^ and 2.2 × 10^–31^ cm^6^ s^−1^, respectively^19^. The bimolecular radiative coefficient is also taken as 9.5 × 10^–15^ cm^3^ s^−1^^19^.

The thickness of the doping regions L_i_ can be selected from Fig. 7b in order to increase the conversion efficiency with a saturated ${J}_{sc}$ of 30.91 mA cm^−2^ at L_i_ equals to 800 nm. In this investigation, the thickness of L_p_ and L_n_ is equal to 100 nm. Figure 7c,d present an electrical comparison between three different embedded dielectric elements with the planer solar TF C-Si-SC. The dimensions of embedded single NW and two nanowires are mentioned in Table 1. The calculated ${J}_{sc}$, the open-circuit voltage (${V}_{oc}$), PCE, and the fill factor (F.F) for the three embedded elements are listed in details in Table 2.

**Table 2:** The J–V characteristics of the TFC-Si-SCs with different embedded dielectric elements.

Dielectric element	V_oc_ (V)	${\mathrm{J}}_{\mathrm{sc}}$(mA cm^−2^)	PCE (%)	F.F (%)
Planer	0.57	18.425	8.58	82
Sphere	0.584	20.44	10.1	82.52
Single NW	0.614	28.3	14.48	83.3
Two NWs	0.623	30.91	15.91	83.01

The enhancement in J–V characteristics for the proposed design with single NW and two NWs can be attributed to the absorption enhancement shown in Fig. 4c. Accordingly, it is expected that the (J–V) results of the proposed design show better electrical performance than that of others. It may be seen from ${J}$ values presented in Fig. 7c that the reported design with embedded two NWs and single NW have higher ${J}_{sc}$ than that of nanosphere and planer based TF C-Si-SC which are 30.91, 28.3, 20.44, and 18.425 mA cm^−2^, respectively. The difference between the values of ${J}_{ph}$ and ${J}_{sc}$ values is attributed to the recombination loses due to Shockley–Read–Hall recombination, radiative recombination, and Auger recombination^12^. It is also evident from Fig. 7c that there is a slight improvement in ${V}_{oc}$, which is mainly determined by the internal dark-current response owing to the increase of ${J}_{sc}$. As for power conversion efficiency (PCE), it can be shown from Table 2 and power density curves in  Fig. 7d that the proposed designs with dielectric two embedded NWs and single NW have better power conversion efficiencies than that of the SiO_2_ nano-sphere and planer based C-Si TFSC with 15.91%, 14.48%, 10.1%, and 8.58%, respectively.

**Figure 8:**
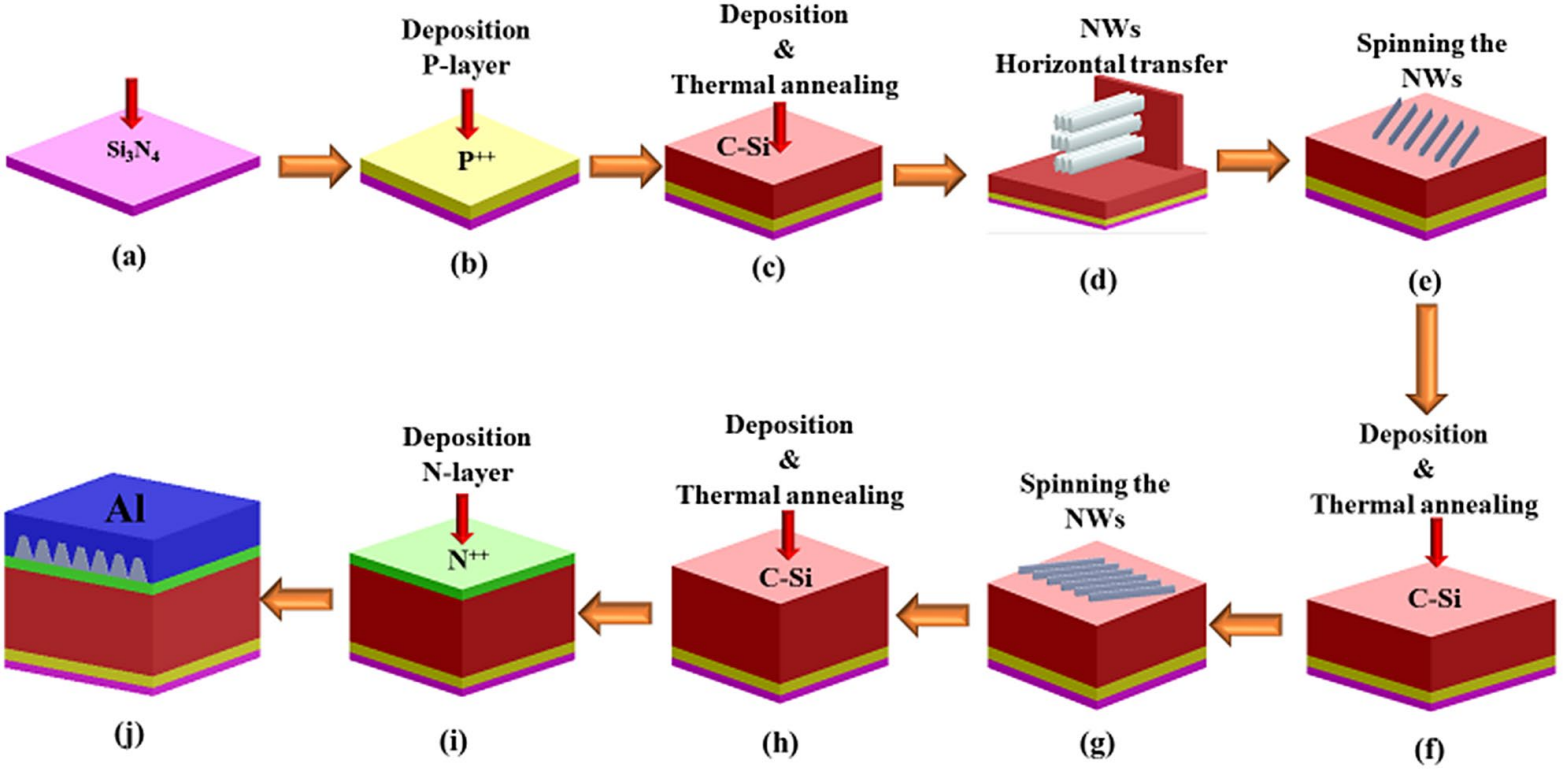
The deposition of (**a**) Si_3_N_4_ and then (**b**) P++ doped Si layers; (**c**) deposition and annealing processes to form the C-Si; (**d**) etched substrate is vertically pressed onto the target substrate; (**e**) the SiO_2_ NWs are transferred onto the target substrate; (**f**) deposition of the second C-Si layer; (**g**) the second batch of SiO_2_ NWs is transferred onto the target substrate; (**h**) the third layer of C-Si is then deposed and annealed; (**i**) deposition of the n++ layer; and (**j**) transfer of the aluminum grating layer. This image is created by Lumerical 2020a, FDTD Solver Version 8.23.2305, https://www.lumerical.com (license number—12802) released to Zewail City of Science and Technology, Giza, Egypt.

The fabrication of the suggested design can be achieved by using physical vapor deposition, atomic chemical vapor deposition, and wet etching process according to the following steps shown in Fig. 8. First, a Si_3_N_4_ layer is deposited with a thickness of 75 nm using atomic chemical vapor deposition. A P-type silicon substrate is exposed to ammonia and hexamethyldisiloxane (HMDSO) gases with flow rates of 80 and 40 SSCM, respectively^20^. A deposition time of 15 min and temperature of 825 °C are needed to obtain a silicon nitride film of 75 nm thickness^20^. Then, a thin layer of silicon is deposited with a thickness of 100 nm at 1000 °C using dichlorosilane (DCS) or Silane (SiH_4_) and diborane (B_2_H_6_) to obtain a heavily doped p-type silicon layer with doping concentration 5 × 10^17^ cm^−3^^21,22^. Such a layer is then annealed in vacuum at a temperature of 1000 °C for crystallization as shown in Fig. 8b^21–26^. Figure 8c shows the deposition of the second silicon layer with a thickness of 236 nm on p-type silicon and Si_3_N_4_ layer at a temperature of 690 °C. The substrate is annealed in vacuum at 1000 °C for 30 min to achieve fully crystalline silicon film^23^. Next, photolithography process is used to make an isotropic etching of the required cleavages to anchor the first layer of NWs^27^. The NWs can be fabricated by using metal-assisted chemical etching (MACE) method^27,28^. The Si NWs are subjected to thermally oxidizing flow to form the SiO_2_ NWs. After that, the SiO_2_ NWs are horizontally transferred by pressing NWs substrate vertically onto the <110> crystalline silicon substrate as shown in Fig. 8d^27^. The SiO_2_ NWs can be aligned to silicon cleavage through spinning process along the surface of the C-Si layer as revealed from Fig. 8e^27,29^. An additional silicon layer of 100 nm thickness is deposited and annealed to obtain the C-Si layer. Then, the isotropic etching and spinning process are repeated for positioning the second batch of the SiO_2_ NWs.

A layer of Si with thickness of 464 nm is next deposited using argon gas and is annealed in vacuum at 1000 °C for 30 min^23^. Additionally, n-thin layer of silicon is deposited with a thickness of 100 nm at 1000 °C using dichlorosilane (DCS), and phosphine (PH_3_) as N-dopants with doping concentration of 1 × 10^19^ cm^−3^^22^.

Finally, an aluminum layer is deposited on sacrificial silicon substrate^30^ or on polyethylene terephthalate (PET) layer^31^. The grating can be etched using mold-assisted chemical etching to achieve the optimized dimension mentioned in Table 1^32,33^. After that, the aluminum grating layer is transferred to the proposed solar cell using flip transfer method^34^. Such sacrificial layer is later exposed to KOH for removal.

## Conclusion

A novel design of TFC-Si-SC with embedded dielectric NWs is presented in order to improve the light-harvesting efficiency. The performance of the suggested design is optimized using PSO technique to maximize the light absorption through the active layer. The optimized SC offers photocurrent density of 32.9 mA cm^−2^ and 30.2 mA cm^−2^ using single/double NWs, respectively. In addition, corresponding power conversion efficiencies of 14.48% and 15.91%, are achieved. Therefore, this work shows the ability of using volume scattering of embedded dielectric NWs for increasing the light trapping and power conversion efficiencies in TFSC.

## References

[1] Obayya SSA, Areed NFF, Hameed MFO, Abdelrazik MH (2017). Optical nano-antennas for energy harvesting. Renew. Altern. Energy.

[2] Laboratory, N. R. E. Best Research-Cell Efficiency Chart. https://www.nrel.gov/pv/cell-efficiency.html (2019).

[3] Hussein M, Swillam MA, Obayya S, Farahat M, Huffaker DL, Eisele H (2018). Electrical characteristics of silicon nanowires solar cells with surface roughness. Quantum Dots and Nanostructures: Growth, Characterization, and Modeling XV.

[4] Mahmoud AHK (2019). Optoelectronic performance of a modified nanopyramid solar cell. J. Opt. Soc. Am. B.

[5] Abdel-Latif GY, Hameed MFO, Hussein M, Abdel Razzak M, Obayya SSA (2018). Characteristics of highly efficient star-shaped nanowires solar cell. J. Photon. Energy.

[6] Nunomura S, Minowa A, Sai H, Kondo M (2010). Mie scattering enhanced near-infrared light response of thin-film silicon solar cells. Appl. Phys. Lett..

[7] Mopurisetty SM, Bajaj M, Sathaye ND, Ganguly S (2015). Coupled optical and electrical analysis for thin-film solar cells with embedded dielectric nanoparticles. Appl. Phys. Lett..

[8] Yang Z (2016). Broadband and wide-angle light harvesting by ultra-thin silicon solar cells with partially embedded dielectric spheres. Opt. Lett..

[9] Nagel JR, Scarpulla MA (2013). Design principles for light trapping in thin silicon films with embedded dielectric nanoparticles. Prog. Photovolt. Res. Appl..

[10] Nagel JR, Scarpulla MA (2010). Enhanced absorption in optically thin solar cells by scattering from embedded dielectric nanoparticles. Opt. Express.

[11] Lumerical: High-Performance Photonic Simulation Software. www.lumerical.com.

[12] Abdel-Latif GY, Hameed MFO, Hussein M, Razzak MA, Obayya SSA (2017). Electrical characteristics of funnel-shaped silicon nanowire solar cells. J. Photon. Energy.

[13] Obayya SSA, Rahman BMA, El-Mikati HA (2000). Full-vectorial finite-element beam propagation method for nonlinear directional coupler devices. IEEE J. Quantum. Electron..

[14] Obayya SSA (2004). Novel finite element analysis of optical waveguide discontinuity problems. J. Light. Technol..

[15] Obayya SSA, Somasiri N, Rahman BMA, Grattan KTV (2003). Full vectorial finite element modeling of novel polarization rotators. Opt. Quantum Electron..

[16] Palik ED (1985). Handbook of Optical Constants of Solids. Handbook of Optical Constants of Solids.

[17] Liscidini M, Bozzola A, Andreani L (2012). Photonic light-trapping and Lambertian limit in thin film silicon solar cells. Opt. Soc. Am..

[18] Heidarzadeh H, Tavousi A (2019). Performance enhancement methods of an ultra-thin silicon solar cell using different shapes of back grating and angle of incidence light. Mater. Sci. Eng. B Solid-State Mater. Adv. Technol..

[19] Yang Z, Li X, Wu S, Gao P, Ye J (2015). High-efficiency photon capturing in ultrathin silicon solar cells with front nanobowl texture and truncated-nanopyramid reflector. Opt. Lett..

[20] Jhansirani K, Dubey RS, More MA, Singh S (2016). Deposition of silicon nitride films using chemical vapor deposition for photovoltaic applications. Results Phys..

[21] Bailly MS, Karas J, Jain H, Dauksher WJ, Bowden S (2016). Damage-free laser patterning of silicon nitride on textured crystalline silicon using an amorphous silicon etch mask for Ni/Cu plated silicon solar cells. Thin Solid Films.

[22] Chen Y (2017). Nanostructured dielectric layer for ultrathin crystalline silicon solar cells. Int. J. Photoenergy.

[23] Bailey LR (2015). High rate amorphous and crystalline silicon formation by pulsed DC magnetron sputtering deposition for photovoltaics. Phys. Status Solidi Appl. Mater. Sci..

[24] Su C-J, Tsai T-I, Lin H-C, Huang T-Y, Chao T-S (2012). Low-temperature poly-Si nanowire junctionless devices with gate-all-around TiN/Al_2_O_3_ stack structure using an implant-free technique. Nanosc. Res. Lett..

[25] Yokoyama S, Ohba K, Kawamura K, Kidera T, Nakajima A (2001). Low-temperature selective deposition of silicon on silicon nitride by time-modulated disilane flow and formation of silicon narrow wires. Appl. Phys. Lett..

[26] Gui C, Albers H, Gardeniers JGE, Elwenspoek M, Lambeck PV (1997). Fusion bonding of rough surfaces with polishing technique for silicon micromachining. Microsyst. Technol..

[27] Zhang D (2014). Horizontal transfer of aligned Si nanowire arrays and their photoconductive performance. Nanosc. Res. Lett..

[28] Peng K, Yan Y, Gao S, Zhu J (2003). Dendrite-assisted growth of silicon nanowires in electroless metal deposition. Adv. Funct. Mater..

[29] Nagel JR, Scarpulla MA (2013). Enhanced light absorption in thin film solar cells with embedded dielectric nanoparticles: Induced texture dominates Mie scattering. Appl. Phys. Lett..

[30] Levine I (2012). Epitaxial two dimensional aluminum films on silicon (111) by ultra-fast thermal deposition. J. Appl. Phys..

[31] Dunn T (2015). Overprint varnishes and coatings. Flexible Packaging.

[32] Lee W (2010). The anodization of aluminum for nanotechnology applications. JOM.

[33] Lai K-L, Hon M-H, Leu I-C (2011). Fabrication of ordered nanoporous anodic alumina prepatterned by mold-assisted chemical etching. Nanosc. Res. Lett..

[34] Zhang K, Seo J-H, Zhou W, Ma Z (2012). Fast flexible electronics using transferrable silicon nanomembranes. J. Phys. D. Appl. Phys..

